# Risk estimation of SARS-CoV-2 transmission from bluetooth low energy measurements

**DOI:** 10.1038/s41746-020-00340-0

**Published:** 2020-10-06

**Authors:** Felix Sattler, Jackie Ma, Patrick Wagner, David Neumann, Markus Wenzel, Ralf Schäfer, Wojciech Samek, Klaus-Robert Müller, Thomas Wiegand

**Affiliations:** 1 Fraunhofer Heinrich Hertz Institute, 10587 Berlin, Germany; 2 Department of Electrical Engineering and Computer Science, Technische Universität Berlin, 10587 Berlin, Germany; 3 Department of Artificial Intelligence, Korea University, Seoul, Korea; 4 Max Planck Institute for Informatics, 66123 Saarbrücken, Germany

**Keywords:** Viral infection, Risk factors, Computer science

## Abstract

Digital contact tracing approaches based on Bluetooth low energy (BLE) have the potential to efficiently contain and delay outbreaks of infectious diseases such as the ongoing SARS-CoV-2 pandemic. In this work we propose a machine learning based approach to reliably detect subjects that have spent enough time in close proximity to be at risk of being infected. Our study is an important proof of concept that will aid the battery of epidemiological policies aiming to slow down the rapid spread of COVID-19.

## Introduction

Contact tracing is an effective instrument to contain and delay outbreaks of infectious diseases such as the ongoing SARS-CoV-2 pandemic. Individuals that have been in contact with an infected person are identified, asked to remain in quarantine and are being tested. However, manually following contact histories is labor-intensive, slow and incomplete, as chance encounters, e.g. in the public transport, can not be fully reconstructed. The emergence of digital solutions, which automatically reconstruct the duration and proximity of encounters, is highly promising to enhance established manual procedures with speed, efficiency, precision and full coverage of relevant contact history. Ultimately, such proximity tracing technologies have the potential to “reduce transmission enough to achieve *R* < 1 and sustained epidemic suppression, stopping the virus from spreading further”^1^.

Various concepts for proximity tracing have been proposed in the past [e.g. refs. ^2–6^]. Recently, the *Pan-European Privacy-Preserving Proximity Tracing* (see ref. ^7^) and *Decentralized Privacy Preserving Proximity Tracing* (see ref. ^8^) initiatives were launched, both promising to enable proximity tracing in compliance with the European general data protection regulation (GDPR)^9^. Since a large percentage of the world’s population carries smartphones, these approaches make use of the Bluetooth low energy (BLE^10^) technology. BLE is a wireless communication protocol, designed for the energy-efficient transmission of data over the 2.4 GHz licence-free band. Contact advertisements regularly emitted via BLE are used to assess the proximity of encounters. For effectively containing the current SARS-CoV-2 pandemic, it is necessary to reliably translate the BLE signal strength measurements into risk estimates of infection transmission. Different studies have investigated the use of BLE measurements for distance estimation and positioning, see, for instance, refs. ^11–14^. These studies show that accurate distance estimation using BLE is difficult due to alternating advertising channels and multi-path effects. These issues are particularly severe in the complex and unknown 3d environments we encounter in our particular use-case. In this letter, we propose a data driven approach to achieve feasible risk estimates from BLE measurements and show that, despite all of these well-known issues, raw RSSI measurements can be sufficient to provide useful contributions to the epidemiological risk assessment.

**Fig. 1:**
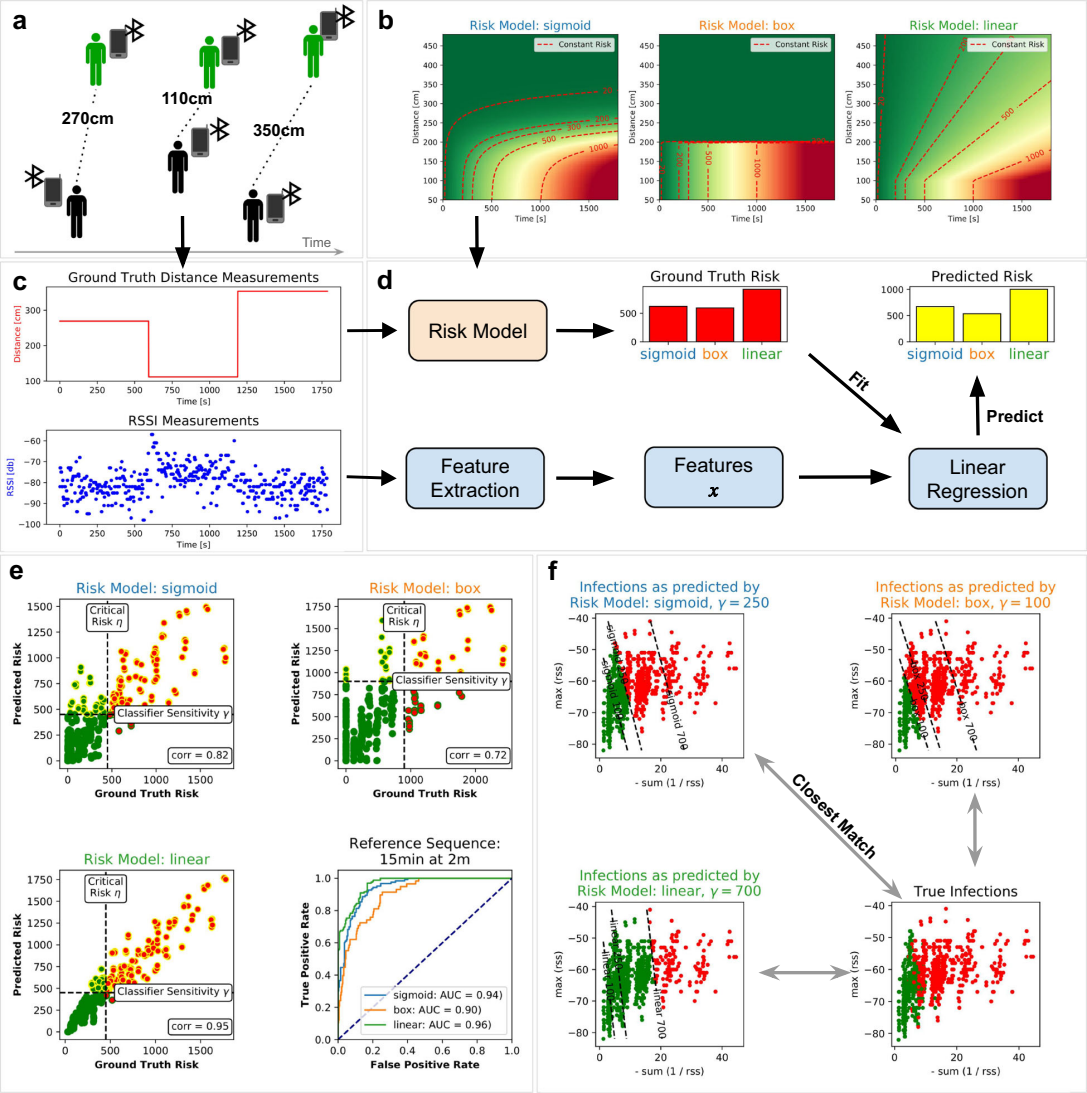
Overview of the proximity tracing concept and results. **a** Typical infection scenario in a public space (e.g. a supermarket), where close contact between an infected and a contact person is established over a long enough period of time. **b** An epidemiological risk function translates a time series of contact distances into infectiousness scores, which are then used to label the encounters in the training data set. **c** Example of a raw RSSI time series of the BLE signal, as well a corresponding contact distances. **d** We train a linear regression model to predict the infectiousness scores obtained from a given risk model. The linear regression receives as input a list of features, which were derived from the raw RSSI data. **e** The predictions of the linear regression model correlate strongly with the ground truth risk (up to 0.95 for the linear risk model). For a fixed critical risk threshold *η* the approach achieves high true positive rates with very few false classifications. **f** To this day only little is known about spreading behaviour of SARS-Cov-2. In this work, we calibrated our epidemiological models according to the latest recommendations of epidemiologists^16^. After large-scale deployment of proximity tracing technologies, it will be possible to compare the predicted infection events with the actually measured ones. This may help to refine epidemiological models.

Figure 1a illustrates a typical infection scenario, which is difficult to manage with manual contact tracing procedures. Here, an infected person enters a public place (e.g. a supermarket) and spends an extended amount of time in close proximity (<2 m) to the contact person. Both factors, namely the contact distance and the contact duration, influence the risk for the contact person of being infected.

Proximity tracing technologies allow to reconstruct such high risk encounters between the infected and contact person, once the former has been tested positive. The infected person is recording anonymous IDs of contact persons within certain critical distance range. These anonymous proximity histories are encrypted and remain on the phone of the infected person at all times. Only if tested positive and upon agreement, the proximity history is analyzed and contact persons with a high risk of being infected can be alerted anonymously. In addition, health authorities can be involved to handle these high risk cases by standard procedures (e.g., test and quarantine the contact persons).

To make this approach practically applicable, i.e., to avoid that every short time or distant encounter raises an alarm, it is crucial to reliably estimate the risk of infection transmission from the BLE signal strength measurements. In this letter we propose techniques to perform this conversion.

## Methods

### Epidemiological risk modeling

We first define an epidemiological model to convert proximity time series to infection risk scores. The models *E* displayed in Fig. 1b implement different non-linear functions to translate time series of proximity values into infection risk scores. For infections transmitted via the droplet route, one usually assumes that the infection risk decreases as the distance *d*_*t*_ between people increases; with some critical distance from which on the risk of being infected becomes vanishingly low^15^. See Supplementary Methods 1 for more details on our choice of epidemiological risk functions. The chosen epidemiological model is then used to label the data needed to train the ML-based infection risk predictor. For that, one integrates the marginal infection risk within the critical distance over the contact duration *T* to obtain an infection risk score

1 $$I=\mathop{\sum }\limits_{t=1}^{T}E({d}_{t}).$$

An encounter between two individuals is considered as “high risk” if the value of *I* exceeds a predefined critical risk threshold *η*. This threshold can either be set either locally, i.e., for each encounter, or globally based on the estimated reproduction rate *R*. For COVID-19 it is assumed that a physical proximity between two people of less than 2 meters over a time period of 900 s (15 min) results in a high risk of being infected^16^. When setting *η* locally, one would use these parameters to determine if an encounter is labelled as “high risk” or not. On the other hand, a globally set critical risk *η*, will label the data such that the number of “high risk” encounters exceeds the expected total number of new cases by a certain safety-margin. See Supplementary Methods 2 for more details on how to chose the value of *η*.

### Machine learning for risk prediction

Finally, we train a linear regression model

2 $$\tilde{I}(x)={w}^{\top }x+b$$

to predict the infection risk score from the measured received signal strength (RSSI) time series of the BLE signal. For simplicity, we do not provide the raw RSSI time series to the ML model, but compute features *x* (sum, mean, max etc.) on it and provide this aggregated information to the model. By thresholding $\tilde{I}$, the output of the linear regression, we obtain a family of classifiers

3 $${\text{risk}}_{\gamma }(x)=\left\{\begin{array}{ll}^{\prime\prime} {\text{high}}\, {\text{risk}}\,^{\prime\prime} &\,{\rm{if}}\,\,{\tilde{I}}(x)\,>\,\gamma \\ ^{\prime\prime} {\text{low}}\, {\text{risk}}\,^{\prime\prime} &\,{\text{else}}\,\end{array}\right.$$

which allows us trade-off sensitivity and specificity of our predictions. Fig. 1d illustrates the entire training and evaluation pipeline, including ground truth risk estimation, feature extraction and training of the linear regression model. See Supplementary Methods 3 for more details on our machine learning approach.

Figure 1c displays the time series of raw RSSI values from the BLE signal, which the smartphone of the infected person receives from the smartphone of the contact person. Although there is high variability in the RSSI values caused by complicated multi-path effects and alternating advertising channels, it is still possible to reliably decide whether or not the infection risk *I* exceeds a certain threshold, as shown in our real-world experiments performed with 48 participants (see Supplementary Note 1 for the details on the experimental setup).

### Experimental evaluation and discussion

Figure 1e, compares the ground truth risk, as computed from the time series of ground truth distances, with the predicted risk, estimated from the Bluetooth signal strength data, for 392 contact episodes from a holdout validation set. As we can see, our machine learning based approach, is able to achieve correlation numbers of up to 0.95 for the linear infection risk model. We compute the critical risk threshold *η* by inserting the reference sequence *d*^ref^, with

4 $${d}_{t}^{{\mathrm{ref}}}\equiv 200\,{\mathrm{cm}}\,{\rm{and}}\ \ {T}^{{\mathrm{ref}}}=900\,{\mathrm{s}}$$

into the different risk models. By varying the classifier sensitivity *γ*, we can trade-off the number of correct and false alarms. The resulting receiver operating characteristic (ROC) curve of the real-world experiment displayed in Fig. 1e shows that high true positive rates can be achieved with relatively few false classifications. Note that these ROC curves depend on the data labeling procedure, i.e., the epidemiological model and the threshold *η*. Here we used the assumed parameters for COVID-19, namely distance <2 m and exposure time >15 min^16^. We provide mean and maximum RSSI value as well as the number of received Bluetooth beacons as features to the linear regression model; results with other features derived from the RSSI time series can be found in Supplementary Fig. 1. The AUC (area under the ROC curve) value of the predictor is found to be larger than 0.9 for all investigated epidemiological models. For the linear model AUCs of up to 0.96 were obtained. The prediction task becomes slightly more difficult for the box and sigmoid models, which assign only negligible risk to encounters above a certain distance. The repetition of this analysis on data recorded on another day led to very similar performance results, demonstrating the reliability of the proposed approach (see Supplementary Table 1).

An important open question is how in detail the distance and duration of a contact to an infected person relate to the risk of contracting Sars-Cov-2. Investigating this relationship in experiments in a controlled environment is morally questionable, since it puts the health of test subjects at risk. Our approach has the potential of discovering the true relationship between contact proximity and infection risk, without putting lives at risk.

Once the true infection events will be observed (given data donations and consent of all users involved), a large record of RSSI time-series with associated ground-truth risk labels will be available. By minimizing the prediction error w.r.t. these true risk labels over the set of risk models *E* and classifier thresholds *γ*, we will be able to identify the true relationship between proximity and infection risk, which will help further improving our risk assessment.

This idea is illustrated in Fig. 1f, which displays RSSI sequence data along with the classification decisions of linear classifiers, that were trained to match the predictions of three different epidemiological models. Every RSSI sequence is represented as a dot and we display only two features of every RSSI sequence, the maximum and the sum of the negative inverse RSSI values.

In this letter we have proposed an approach to reliably detect subjects that have spent enough time in close proximity to be at risk of having contracted an infectious disease. Thus our study is an important proof of concept that will aid the battery of epidemiological policies aiming to slow down the rapid spread of COVID-19. Note that while we have assumed the standard modeling of viral spread with the currently agreed on parameters (distance <2 m and exposure time >15 min, see ref. ^16^), it may in fact be conceivable that these parameters are not chosen conservatively enough in the light of recent results on contagious droplet spreading across larger distances rsp. in aerosols (see e.g. ref. ^17^) and moreover the improved binding affinity of SARS-CoV-2^18^. Clearly, once proximity tracing technologies will be rolled out for the broad population, then transmission events will become available that will provide evidence for the true epidemiological modeling assumptions. With that we could find out whether the current risk assessment is conservative enough or whether indeed social distancing would need to be increased further.

Finally, it is important to emphasize that there are technical limitations of the BLE technology which make it impossible to detect certain epidemiologically relevant events. For instance, it is impossible to detect, using BLE measurements, whether a contact tracing app user is wearing a face-mask or not. To further improve the results, it could therefore be helpful to consider additional sources of data, like user questionnaires or the phones GPS and gyroscope sensor. These are interesting directions of future research.

### Reporting summary

Further information on research design is available in the Nature Research Reporting Summary linked to this article.

## Supplementary information

Supplementary Information

Reporting Summary

## Data Availability

The data that support the findings of this study are publicly available at https://github.com/felisat/ble-proximitiy-tracing.

## Code availability

The code that was used to pre-process and analyse the data is available from the corresponding author upon request.
