# Supplementary material for: Risk estimation of SARS-CoV-2 transmission from bluetooth low energy measurements
Source: NPJ Digit Med. 2020 Oct 6;3:129. doi: 10.1038/s41746-020-00340-0 (PMC7538938; doi:10.1038/s41746-020-00340-0)
Supplement: Supplementary file 1 — Supplementary Information [file 41746_2020_340_MOESM1_ESM.pdf]

Risk Estimation of SARS-CoV-2 Transmission from Bluetooth Low Energy Measurements
# – Supplementary Materials –

Felix Sattler[1], **Jackie Ma**[1], **Patrick Wagner**[1,2], **David Neumann**[1], **Markus Wenzel**[1], **Ralf Schäfer**[1], **Wojciech Samek**[1,✉], **Klaus-Robert Müller**[2,3,4,✉], **and Thomas Wiegand**[1,2,✉]

[1]Fraunhofer Heinrich Hertz Institute, 10587 Berlin, Germany
[2]Department of Electrical Engineering and Computer Science, Technische Universität Berlin, 10587 Berlin, Germany
[3]Department of Artificial Intelligence, Korea University, Seoul, Korea
[4]Max Planck Institute for Informatics, 66123 Saarbrücken, Germany
✉{wojciech.samek, thomas.wiegand}@hhi.fraunhofer.de, klaus-robert.mueller@tu-berlin.de

Supplementary Methods 1: Epidemiological Models

In our experiments we use three different epidemiological models to convert the proximity values into infectiousness scores

$$E_{linear}(d) = \begin{cases} 1 & \text{if } d[cm] < 100 \\ \frac{100}{d[cm]} & \text{if } 100 < d[cm] < 500 \\ 0 & \text{else} \end{cases} \tag{1}$$

$$E_{box}(d) = \begin{cases} 1 & \text{if } d[cm] \leq 200 \\ 0 & \text{else} \end{cases} \tag{2}$$

$$E_{sigmoid}(d) = \left(1 + \exp\left(\frac{d[cm] - 200}{30}\right)\right)^{-1}, \tag{3}$$

where d is the contact distance measured in cm. All three models are monotonically decreasing functions of the distance to account for the fact that the infectiousness score decreases with increasing distance. Note, that in the absence of clinical studies on the infectiousness of Sars-Cov-2, these proposed epidemiological risk functions merely represent plausible options for the relationship between the distance to an infected individual and the infection risk, while the true relationship is unknown. Investigating this relationship further is an interesting direction of future research.

The main use of epidemiological models in our experiments is to generate ground truth labels for our data, which consists of a time series of RSSI values and corresponding distances (the latter is not available in real settings). To generate the labels, we integrate the infectiousness scores over the contact time according to the equation

$$I(d_1, .., d_T) = \sum_{t=1}^{T} E(d_t). \tag{4}$$

Supplementary Methods 2: Local and Global Risk Thresholds

For every epidemiological model E there exists a *reference* proximity, from which on no infection is expected. For instance, for COVID-19 it is assumed that a physical proximity between two people of less than 2 meters over a time period of 900 seconds (15 minutes) results in a high risk of being infected [1]. Inserting the reference sequence d^{ref}, with

$$d_t^{ref} \equiv 200cm \text{ and } T^{ref} = 900s \tag{5}$$

into equation (4) results in a *local threshold*

$$\eta = I(d^{ref}) = \sum_{t=1}^{T^{ref}} E(d_t^{ref}) \tag{6}$$

By selecting the epidemiological model and the infectiousness threshold we can determine, which time series of distance measurements should be considered dangerous and which should not:

$$\text{HighRisk}(d_1, .., d_T) = \begin{cases} True & \text{if } I(d_1, .., d_T) > \eta \\ False & \text{if } I(d_1, .., d_T) \leq \eta \end{cases} \tag{7}$$

An alternative approach is to label the data with a *global threshold* based on an estimate of the current reproduction number R. If N_{inf} people are currently infected, then we can expect that each infected individual will pass on the disease R times such that the total number of new infections is at the order of

$$N_{new} = RN_{inf} \tag{8}$$

We can then chose η in a way so that the number of high risk encounters matches the expected number of new infections, i.e.,

$$\sum_{d \in D} \mathbb{1}_{\text{HighRisk}(d)} = N_{new} \tag{9}$$

or exceeds it by a certain safety margin. Hereby D is the total number of recorded proximity histories.

Supplementary Methods 3: Infection Risk Estimation as a Regression Problem

Given an epidemiological model and the true distances we can label encounters into "high risk" and "low risk". Since the true distances are not available in real settings, we aim to train a machine learning model to predict these labels from the raw RSSI measurements of the BLE signal (For practical reasons we resampled the RSSI values to 1Hz.). To simplify the learning task, we extract features from the RSSI data and provide them as input to the ML algorithm. In particular, we tested the following three feature sets:

1. **sum**: Total sum of received RSSI values resulting in one-dimensional features. We take this as our baseline.
2. **dur_max_mean**: Duration, maximum and mean of received RSSI values resulting in three-dimensional features. (In practice one might want to truncate the duration feature to avoid false alarms for long-duration, high-distance encounters.).
3. **freq**: Amplitudes of first 30 frequencies of received RSSI values resulting in 30-dimensional features.

We input these features x into a linear regression model in order to obtain a predicted "risk" score:

$$\tilde{I}(x) = w^{\top}x + b \quad (10)$$

The input to the linear regression thus comprises a vector of parameters w, a bias term b and a vector of extracted features x. The resulting predicted risk score is then compared to a threshold γ to obtain a binary classification label:

$$\text{risk}_{\gamma}(x) = \begin{cases} \text{"high risk"} & \text{if } \tilde{I} > \gamma \\ \text{"low risk"} & \text{else} \end{cases} \quad (11)$$

If the predicted risk exceeds the threshold, the encounter which created the sequence of RSSI measurements is considered "high risk".

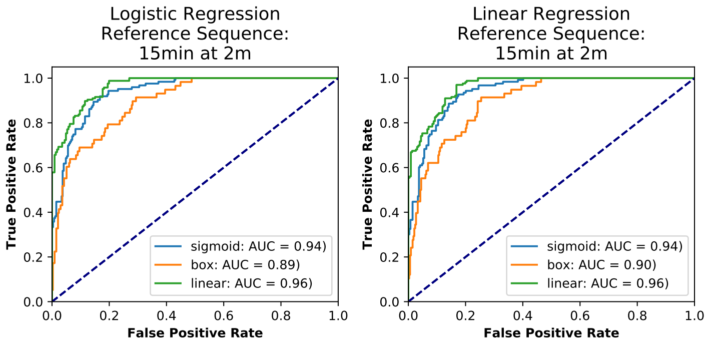

Figure 1: Results achieved, when directly classifying high/ low risk using a Logistic Regression (left) and the Linear Regression approach presented in the paper (right).

An alternative to using a linear regression for risk prediction, would be to directly predict the binary risk label using a logistic regression. As can be seen in Fig 1, this approach achieves similar results, but the predictions are less amenable for interpretation and also directly depend on the choice of the critical risk threshold η, which is why we ultimately decided to use a linear regression model.

Instead of feeding handcrafted features into a linear model, one could also use end-to-end machine learning models like recurrent neural networks [2] or transformers [3]. With the relatively small amounts of data available from our real world experiment, we were not able to achieve comparable results with these more complex models. However, once larger amounts of data will be available from data donations, this approach might become viable.

Supplementary Note 1: Experiment Setup and Data Preparation

Real-World Experiment

A measurement campaign was performed to test and validate the proposed infection risk estimation model. This section describes the setup of the experiment.

The measurements on the 1st of April and the 7th of April were performed using 48 Samsung A40 smartphones of the same type that were carried by 48 protected soldiers, respectively. In the experiments performed on the 14th of April 37 test subjects participated and the following devices were used:

- 29 Samsung SM-A405FN
- 2 Samsung SM-G965F
- 1 Samsung SM-G975F
- 1 Samsung SM-A505FN
- 1 Samsung SM-A750FN
- 1 Samsung SM-J530F
- 1 HUAWEI VOG-L29
- 1 Samsung SM-A520F

Tests were carried out at five different locations within the Julius Leber barracks in Berlin. There were three rooms within a conference center and two outdoor locations, with ten subjects each. All test subjects were equipped with face masks so that there was no risk of infection.

The floor of the test areas was marked (Fig. 2). These markings consisted of a 5 m x 5 m grid with lines spaced 50 cm apart. From the starting point (box within a box) to the ending point (multiplication sign), the test subjects had to walk through markings and stay on each marker for a predetermined amount of time. The traversal through the grid was repeated four times, with identical movement pattern and the stay time varying between 2, 4, 6, and 10 minutes. During the runs, the test subjects were instructed not to move too much, to hold the positions of the mobile phones relatively stable, and to stand within the square. The markings are numbered on the green path from 1 to 9 and on the black path from 2 to 10 (Fig. 2, right). Two cameras were installed at each location to video record the test so that the exact locations of the test subjects could be checked after the test.

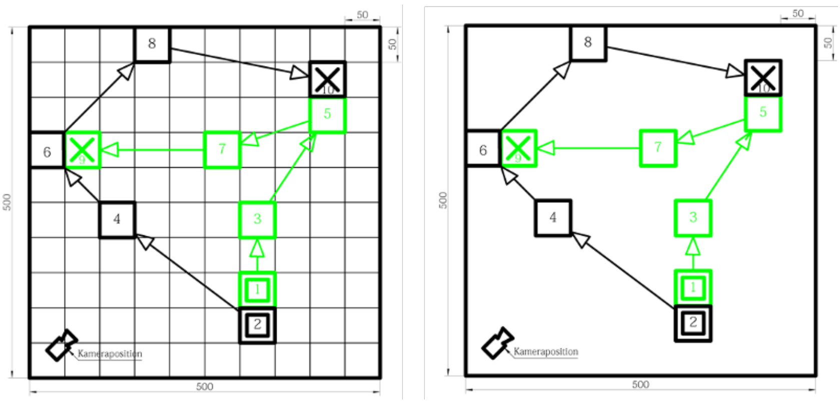

Figure 2: Test pattern on the floor of the five test areas (left with grid, right without grid).

Data and Preprocessing

RSSI data was collected via a prototype of the PEPP-PT App. In order to compute the Fourier coefficients for the `freq` feature, the RSSI data - recorded at a random and potentially varying frequency between 0.1 Hz and 10 Hz - was re-sampled to 1Hz, by setting all undersampled RSSI measurements to the next recorded value ("backward fill"). Ground truth distance data was derived from the predefined movement pattern on the grid. The labeling was additionally verified with the help of video footage that was taken at the test area. For every pair of soldiers we collected 8 data points, one for each of the four traversals through the grid, times two, since every device can act as a receiver and as a sender. Each data point comprised of two aligned sequences:

- A time series of distances $d_t, t = 1, .., T$ (from which the ground truth risk can be derived).
- A time series of BLE RSSI values $RSS_t, t = 1, .., T$, recorded by mobile phones held by the soldiers.

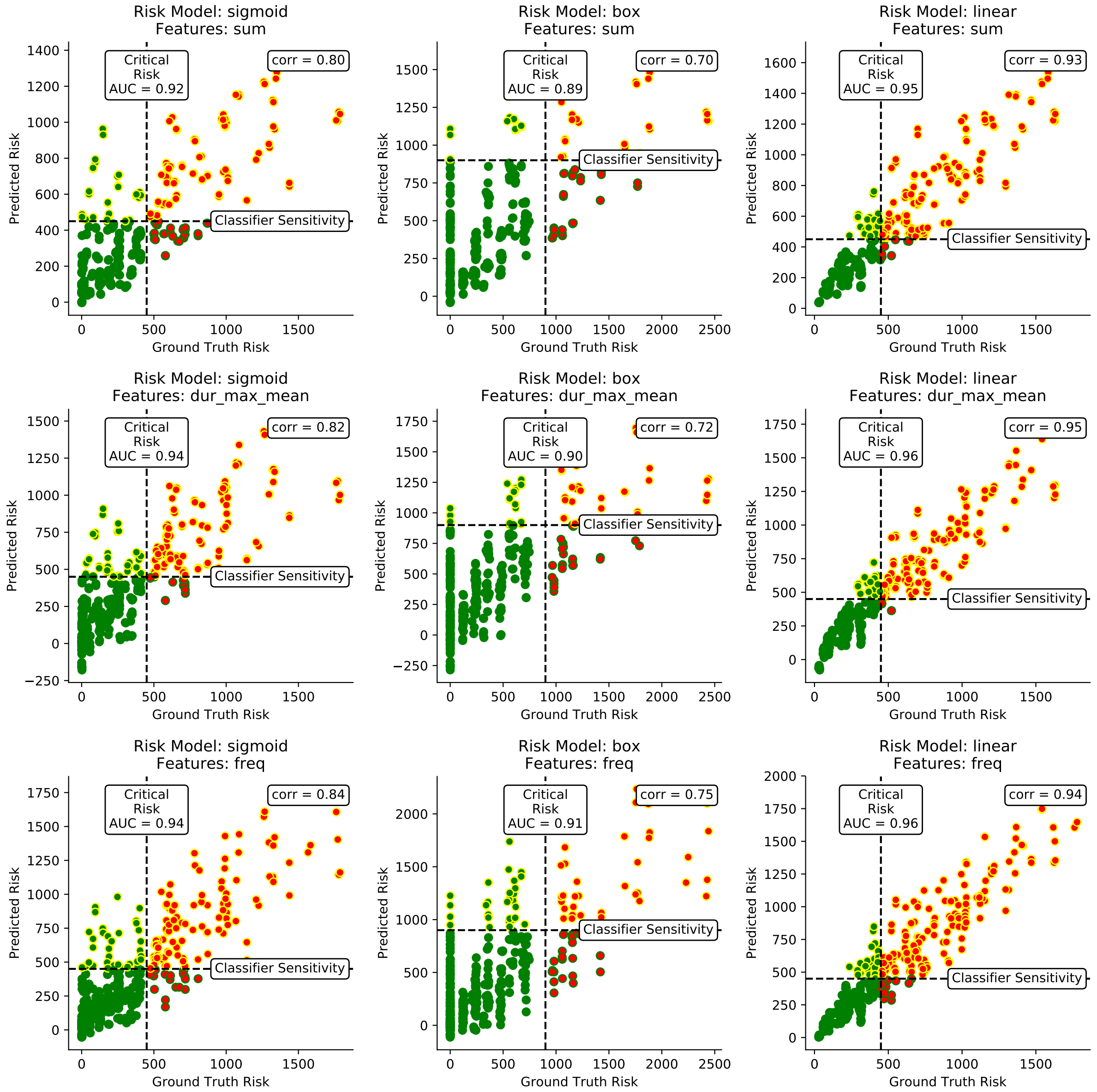

Figure 3: Ground truth risk vs predicted risk for different epidemiological risk models and combinations of features supplied to our machine learning model.

The data RSSI measurements recorded by the different smartphone models on the 14th of April were additionally calibrated by adding two device-specific correction terms, one for the receiver device ($\Delta_{R,X}$) and one for the transmitter device ($\Delta_{T,Y}$) according to

$$RSSI_{X,Y}^{corrected} = RSSI_{X,Y}^{received} + \Delta_{R,X} + \Delta_{T,Y}. \tag{12}$$

These correction terms were pre-calculated according to the make and model of the device.

Training and Testing Data:

For training and testing, the time series data was separated into two folds according to the room in the test area in which the data was collected. Data collected in rooms 1 and 2 (indoor) and room 4 (outdoor) was combined in the training set. Data collected in rooms 3 (indoor) and 5 (outdoor) was combined in the validation set. This methodology ensures that all data points generated from one pair of devices end up in the same split. In previous tests multiple

	Linear			Box			Sigmoid		
	`dur_max_mean`	`sum`	`freq`	`dur_max_mean`	`sum`	`freq`	`dur_max_mean`	`sum`	`freq`
01.04.	**0.971**	0.948	0.956	0.85	0.839	**0.861**	**0.889**	0.87	0.881
07.04.	**0.976**	0.953	0.966	**0.881**	0.849	0.872	**0.921**	0.882	0.893
14.04.	**0.975**	0.893	0.918	**0.853**	0.758	0.781	**0.889**	0.789	0.814

Table 1: Comparison of the results on data recorded on three different days. Only the feature set `dur_max_mean` is robust to the changes in testing environment that occurred during the third measurement campaign on April 14th.

combinations of indoor and outdoor rooms were tested to investigate possible covariate shift between indoor and outdoor scenarios. No significant effects could be detected, therefore the aforementioned mixed split was used.

Supplementary Figure 1: Results

We trained a machine learning model to predict the ground truth risk, by only using features extracted from the RSSI time series data $RSS_1, .., RSS_T$. Since the labels are not balanced (i.e. there are more negative than positive events), we use area under the ROC (receiver operating characteristics) curve (AUC) metric to evaluate the performance of our model. The AUC metric is a measure for how well the data can be separated using our classifier. An AUC value of 0.5 indicates no predictive power and 1.0 indicates perfect predictive power.

The obtained results are presented in Fig. 3. The columns correspond to different epidemiological models, namely (`linear`, `box`, `sigmoid`), whereas the rows represent different combinations of features which we feed into the linear regression. Given the critical risk threshold derived by applying the respective risk model to the reference sequence, we display the achieved AUC for every combination of risk model and feature combination.

An encounter between two individuals is labeled as "high risk" if the value of I exceeds a predefined critical risk threshold η. This threshold can either be set locally, i.e., for each encounter, or globally based on the basic reproduction rate

R_0.

Supplementary Table 1: Follow-up Study

In order to evaluate the reliability of our results, we tested the model which we trained on data from the 1st of April on data recorded with the same experimental setup, but on a different dates (7th April 2020 and 14th April 2020). In the experiments conducted during the 14th of April, participants were using different smart phone models and the phone holding positions were varied ("hand", "ear", "pocket"). Table 1 compares the AUC values of the two measurement campaigns for the three epidemiological models (`linear`, `box`, `sigmoid`) and three sets of features (`sum`, `dur_max_mean`, `freq`). As can be seen, the performance of the proposed infection risk estimation method is comparable for the experiments conducted on the 1st of April and the 7th of April. For the experiments conducted on the 14th of April however the feature set `dur_max_mean` distinctively outperforms all other tested feature combinations. Evidently this combination of features is able to approximate the ground truth risk in a more robust way than the other investigated feature combinations. Note that maximum-filtering is also a commonly used technique in BLE indoor positioning application (see e.g. [4]), which might explain the good performance of this particular feature set.

References

[1] European Centre for Disease Prevention and Control. Contact tracing: public health management of persons, including healthcare workers, having had contact with covid-19 cases in the european union – second update, 2020.

[2] Hochreiter, S. and Schmidhuber, J. Long short-term memory. *Neural Computation*, 9(8):1735–1780, 1997.

[3] Devlin, J., Chang, M.W., Lee, K., and Toutanova, K. Bert: Pre-training of deep bidirectional transformers for language understanding. In *Proceedings of the 2019 Conference of the North American Chapter of the Association for Computational Linguistics: Human Language Technologies, Volume 1 (Long and Short Papers)*, pages 4171–4186, 2019.

[4] Cantón Paterna, V., Calveras Auge, A., Paradells Aspas, J., and Perez Bullones, M.A. A bluetooth low energy indoor positioning system with channel diversity, weighted trilateration and kalman filtering. *Sensors*, 17(12):2927, 2017.
